# Seasonal variation of dung-associated arthropods in cattle pastures of Terceira Island (Azores): a year-round, event-based dataset

**DOI:** 10.3897/BDJ.14.e186171

**Published:** 2026-04-09

**Authors:** Sophie Wallon, Abrão Leite, Almudena Duenas-Rojas, Eva Cuesta, Ana M. C. Santos, Sébastien Lhoumeau, Paulo A. V. Borges

**Affiliations:** 1 University of Azores, CE3C—Centre for Ecology, Evolution and Environmental Changes, Azorean Biodiversity Group, CHANGE —Global Change and Sustainability Institute, School of Agricultural and Environmental Sciences, Rua Capitão João d’Ávila, Pico da Urze, Angra do Heroísmo, Azores, Portugal University of Azores, CE3C—Centre for Ecology, Evolution and Environmental Changes, Azorean Biodiversity Group, CHANGE —Global Change and Sustainability Institute, School of Agricultural and Environmental Sciences, Rua Capitão João d’Ávila, Pico da Urze Angra do Heroísmo, Azores Portugal; 2 Rua Fernando Pessoa, nº99 R/C DTO 2765-483, Estoril, Portugal Rua Fernando Pessoa, nº99 R/C DTO 2765-483 Estoril Portugal; 3 Centro de Investigación en Biodiversidad y Cambio Global (CIBC-UAM), Universidad Autónoma de Madrid, 28049, Madrid, Spain Centro de Investigación en Biodiversidad y Cambio Global (CIBC-UAM), Universidad Autónoma de Madrid, 28049 Madrid Spain; 4 Terrestrial Ecology Group (TEG-UAM), Departamento de Ecología, Universidad Autónoma de Madrid, 28049, Madrid, Spain Terrestrial Ecology Group (TEG-UAM), Departamento de Ecología, Universidad Autónoma de Madrid, 28049 Madrid Spain; 5 IUCN SSC Atlantic Islands Invertebrate Specialist Group, Angra do Heroísmo, Azores, Portugal IUCN SSC Atlantic Islands Invertebrate Specialist Group Angra do Heroísmo, Azores Portugal; 6 IUCN SSC Monitoring Specialist Group, Angra do Heroísmo, Azores, Portugal IUCN SSC Monitoring Specialist Group Angra do Heroísmo, Azores Portugal

**Keywords:** dung beetles, Scarabaeidae, Staphylinidae, Hydrophilidae, cattle-grazed grasslands, baited pitfall traps, seasonality

## Abstract

**Background:**

Dung-associated arthropods, particularly dung beetles (Scarabaeidae), together with rove beetles (Staphylinidae) and water scavenger beetles (Hydrophilidae), support key ecosystem functions in cattle-grazed landscapes, including dung removal, nutrient cycling, soil aeration and pest suppression. Their activity is strongly seasonal in temperate systems, driven by temperature and moisture and can be further reshaped by pasture management (e.g. changes in grazing regimes and dung availability). Oceanic islands add an important perspective because species pools are typically smaller and often dominated by introduced taxa, potentially altering phenology and dominance patterns across the year. However, year-round, standardised, event-based datasets for dung-associated arthropod assemblages remain scarce for oceanic islands, limiting robust comparisons amongst guilds, sites and management regimes and reducing our ability to benchmark seasonal windows of activity under climate variability and land-use change.

**New information:**

We provide a one-year, monthly, standardised dataset of dung-associated arthropods from two cattle pastures on Terceira Island (Azores, Portugal), spanning October 2022 to September 2023. Sampling used dung-baited pitfall traps (four traps per site per month, deployed for 2–4 days) at a low-elevation pasture (“University of Azores Campus”, 41 m) with seasonal cessation of grazing during summer maize cultivation and a mid-elevation pasture (“University of Azores Granja”, 380 m) grazed year-round. The published Darwin Core Archive includes an Event core (96 sampling events) and an Occurrence extension (1,701 occurrence records), with associated metadata including minimum and maximum temperatures per event. In total, 13,882 individuals, belonging to four classes, 16 orders and 61 families, were assigned to 175 morphospecies; 143 morphospecies (12,865 specimens) were identified to full scientific name.

The annual series documents contrasting seasonal dynamics amongst focal dung-associated beetle groups: Scarabaeidae show a short May–July peak at Granja, but a longer, irregular activity period at Campus; Staphylinidae remain active most of the year with site-specific peaks; and Hydrophilidae display a strong late spring–summer pulse at Granja, but a weaker, more prolonged pattern at Campus.

## Introduction

Dung-associated arthropods, most notably dung beetles (Scarabaeidae, Aphodiidae and Geotrupidae), but also many rove beetles (Staphylinidae) and water-scavenger beetles (Hydrophilidae), are particularly vital in agricultural landscapes, where dung livestock represents a significant organic input that requires efficient decomposition (dung removal) to maintain pasture health and productivity (Pecenka and Lundgren 2018). These insects play crucial ecological roles in nutrient cycling, soil aeration, pest suppression and secondary seed dispersal within pastoral ecosystems (Medina and Lopes 2014, deCastro‐Arrazola et al. 2022); indeed, through dung burial and relocation, these insects enhance soil fertility, improve water infiltration and facilitate seed dispersal, thereby sustaining both plant productivity and ecosystem resilience (Beynon et al. 2015, Slade and Roslin 2016, Barragán et al. 2022, Anderson et al. 2024) .

Given their reliance on dung resources, dung-associated arthropod activity is closely linked to the presence of vertebrates and the environmental conditions that regulate decomposition processes. Their abundance and diversity are strongly influenced by seasonal changes in temperature, humidity and precipitation (Leandro et al. 2023, Ndozi et al. 2025) which determine not only the availability of dung, but also the physiological performance and reproductive cycles. Consequently, examining the seasonal dynamics of dung-associated arthropods can provide valuable insights into how environmental variability shapes their ecological functions throughout the year. For example, dung beetles illustrate how changes in temperature and rainfall influence activity patterns, reproduction and resource use (Lobo and Cuesta 2021). Such knowledge is particularly relevant under ongoing climate change, as shifts in temperature and rainfall regimes may alter the temporal patterns of dung-associated beetle activity and, in turn, their contribution to ecosystem services (Maldaner et al. 2021, Carreón et al. 2025).

The activity of dung-associated assemblages reflects both natural climatic variations (e.g. changes in weather, moisture or temperature) and management practices (e.g. grazing regimes, dung availability, use of antiparasitic drugs) over short time periods (Hammer et al. 2016, Tonelli et al. 2019, Numa et al. 2020, Ambarlı et al. 2021, Noriega et al. 2023). This makes them sensitive bioindicators of seasonal environmental variation in temperate regions (Halffter and Favila 1993). In such climates, abundance and richness typically rise from mid-spring through summer and decline in winter, with temperatures generally being a stronger driver than rainfall and with marked phenological partitioning amongst species and guilds (Borges et al. 2017, Rebaudo and Rabhi 2018, Lobo and Cuesta 2021). These patterns have been shown repeatedly across temperate grasslands and forests, where species segregate by season and even by diel period, facilitating co-existence and stabilising function (Canziani and González-Vainer 2024). Understanding the phenological patterns and seasonal segregation of these coprophilous communities is critical for predicting ecosystem responses to ongoing climate change and anthropogenic pressures, which significantly alter species' activity patterns and community structure (Wassmer 2020, Asha et al. 2021).

Beyond Scarabaeidae, co-occurring dung-associated guilds can dominate seasonal dynamics. Rove beetles are often numerically abundant predators and parasitoids of dipterans within pats, while hydrophilids contribute substantially to detrital processing, both groups showing pronounced seasonal turnover (Walsh and Posse 2003). Yet these guilds are frequently under-represented in seasonal datasets, which can bias inferences about whole-community function across the year (Walsh and Posse 2003).

Oceanic islands offer an additional lens on seasonality: regional species pools are smaller and often dominated by exotics (Borges et al. 2022), which can amplify dominance and homogenisation, potentially altering the timing and magnitude of ecosystem functions along the annual cycle. In the Azores, where cattle-grazed pastures occupy extensive areas (Almeida et al. 2021), dung beetle faunas are species-poor and largely introduced and first inventories already highlight strong dominance by a few *Onthophagus* species (with ongoing taxonomic clarification of the *O. vacca* /*O. medius* complex and their distinct phenologies) (Duenas-Rojas et al. 2025).

Despite this context, year-round, standardised, event-based datasets for dung-associated arthropod assemblages remain scarce in Atlantic islands (but see Borges et al. (2017)), limiting our ability to: (i) quantify seasonal windows of peak activity across guilds; (ii) test how pasture management (e.g. temporary cessation of grazing) reshapes phenology and (iii) benchmark insular seasonal profiles against continental temperate systems. Recent work on Terceira at two contrasting pastures suggests management can distort the expected spring–summer peak mentioned above, highlighting the need for complete annual series and explicit Darwin Core Event/Occurrence publishing to enable reuse (Borges et al. 2026). Therefore, documenting the seasonal patterns of activity in dung beetles and dung-associated guilds advances ecological understanding and provides a crucial baseline for evaluating the impact of climate variability and human activity on grassland systems.

Here, we provide a one-year, monthly, standardised dataset of dung-associated arthropods from two different cattle pastures on Terceira Island (Azores) (see also Duenas-Rojas et al. (2025)). We sampled with baited pitfall traps, curated records to species or morphospecies with taxonomic quality control and publish the data as a Darwin Core Archive with an Event core and Occurrence extension to facilitate integration with GBIF and comparability with prior datasets on dung-associated arthropods. This contribution complements recent Azorean inventories (e.g. Borges et al. (2022)) and provides a foundation for comparative analyses of seasonal structure, dominance and functional diversity in temperate insular pasture systems.

## General description

### Purpose

The purpose of this dataset is to document the seasonal dynamics of dung-associated arthropod assemblages in pastures on Terceira Island (Azores) over a full annual cycle. The dataset captures temporal patterns in Scarabaeidae (dung beetles), Staphylinidae (rove beetles) and Hydrophilidae (water scavenger beetles), as well as other arthropods sampled, by providing standardised, event-based records of species occurrence and abundance, along with associated metadata on sampling effort, environmental conditions and pasture management.

### Additional information

This study is part of the DUNGPOOL project, which aims to understand how regional species pool size and composition, together with community assembly mechanisms (including priority effects and biotic interactions) and near-term climate warming, shape dung beetle diversity and the ecosystem functions they support in pasturelands. By combining replicated field experiments on the Iberian mainland with parallel studies on three mid-Atlantic islands of the Azores Archipelago (Pico, Faial and Terceira), the project takes advantage of the strong contrast between a species-rich continental pool and the species-poor, largely exotic island assemblages to test biodiversity–ecosystem function (BEF) hypotheses across spatial scales.

## Project description

### Title

Effects of species pool and community assembly processes on dung beetle diversity and ecosystem functions in a warming world (DUNGPOOL)

### Personnel

Principal investigator: Ana M. C. Santos

Fieldwork (site selection and experimental setting): Paulo A.V. Borges.

Fieldwork (authorisation): Azorean Regional Directorate for the Environment (Internationally Recognized Compliance Certificate 28/2022/DRCT and CCIR-RAA/2023/28).

Fieldwork team: Abrão Leite, Paulo A.V. Borges.

Parataxonomist: Abrão Leite.

Taxonomists: Paulo A.V. Borges and Eva Cuesta.

Database management: Paulo A. V. Borges and Sébastien Lhoumeau.

Darwin Core databases: Sébastien Lhoumeau, Paulo A.V. Borges and Sophie Wallon

### Funding

Agencia Estatal de Investigación, Ministerio de Ciencia, Innovación y Universidades (Spain) (PID2021-122380NA-I00); MICIU/AEI/10.13039/501100011033; FEDER, UE. For the Azores work, additional funding was obtained from FCT-UIDB/00329/2020–2024 (DOI 10.54499/UIDB/00329/2020), FCT-UID/00329/2025, Azores DRCT Pluriannual Funding (M1.1.A/FUNC.UI&D/010/2021-2024) and also M1.1.A/FUNC.UI&D/021/2025 [UI&D/GBA/2025].

## Sampling methods

### Study extent

**Figure 1.**
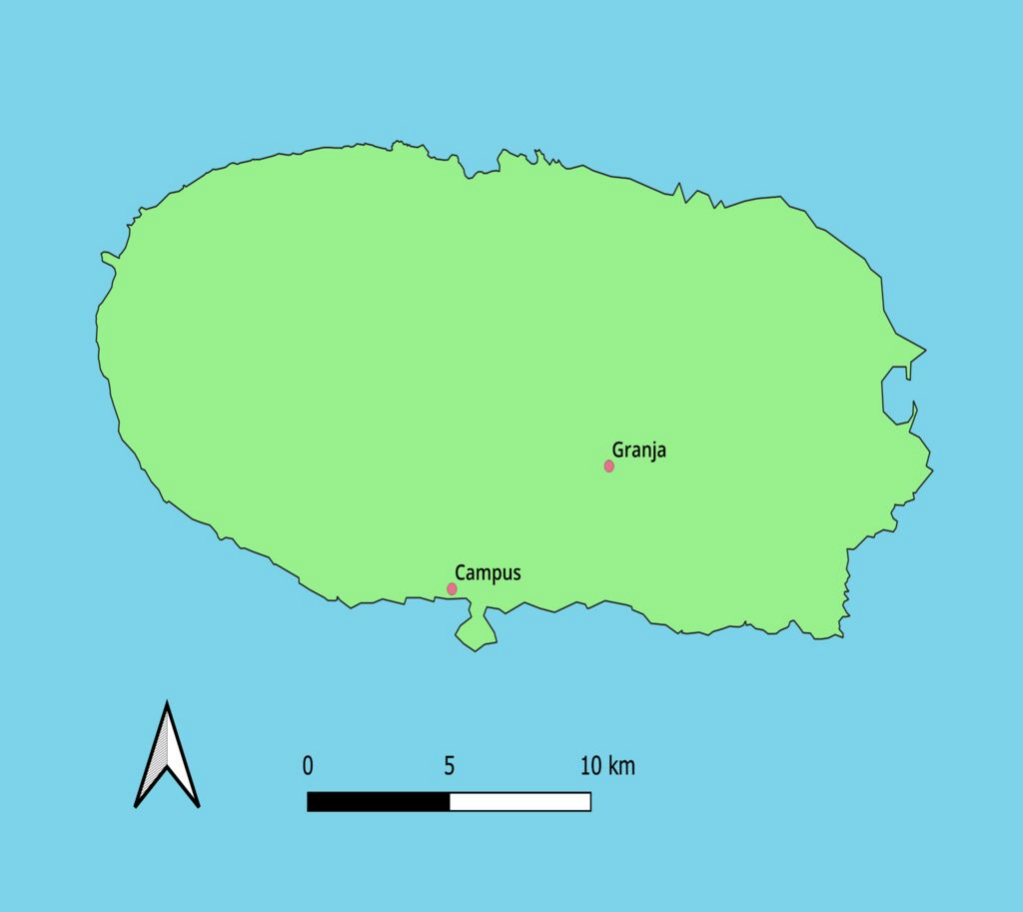
Location of the two sampling sites, Campus and Granja, on Terceira Island in the Azores (Portugal).

This study was conducted from October 2022 to September 2023, in two pasture areas in the island of Terceira (Azores): Campus [Lat: 38.65864 and Long: -27.23516; Elevation: 41 m; mean annual temperature: 16°C] and Granja [Lat: 38.69825 and Long: -27.17149; Elevation: 380 m; mean annual temperature: 14°C] (Fig. 1). The two pasturelands experienced contrasting grazing regimes throughout the year. While cattle at Granja grazed all year round, grazing at Campus ceased during the summer months (June to September) to allow maize to be cultivated.

### Sampling description

**Figure 2.**
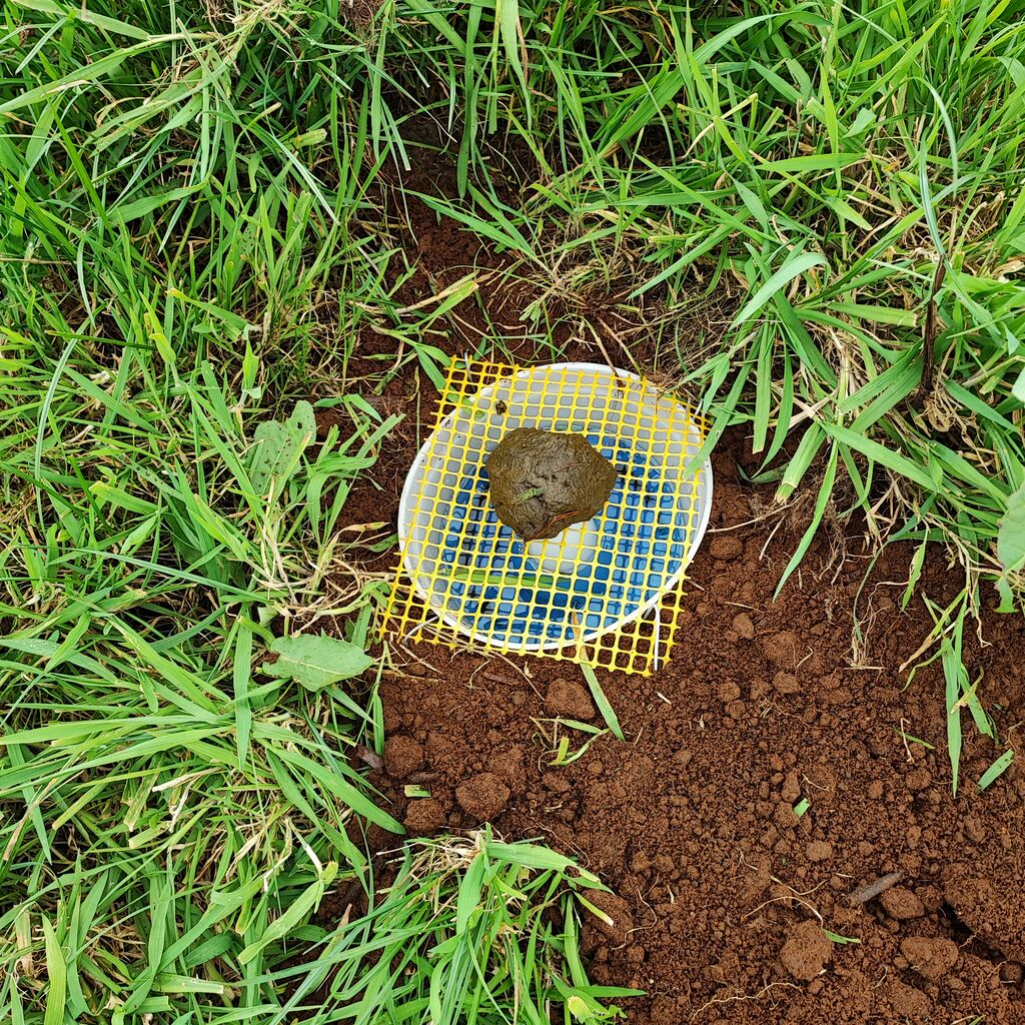
Pitfall trap used to capture the dung associated arthropods (Credit: Paulo A.V. Borges).

Coprophagous insects were sampled using baited G360 traps (Entomopraxis, Barcelona, Spain), a pitfall-type device widely used for collecting dung- and carrion-associated insects. The trap consists of a circular plastic container 20 cm in diameter and 9.5 cm in height, with a central internal protrusion (3.8 cm in diameter) reaching the upper rim, on which a plastic cup containing the bait is placed (Fig. 2). The collecting container was partially filled with a soap-water solution and each trap was baited with 250–300 g of fresh cow dung. To protect the bait and reduce disturbance or removal by mammals, a yellow plastic grid was placed over the trap opening while still allowing access to the target dung-associated insect fauna.

At each pasture, four traps were installed monthly along a transect, with 5 m between consecutive traps and remained active in the field for 2–4 days depending on weather conditions and logistical constraints. In January, traps were exposed for 4 days; from February to September, for 3 days; and in October, November and December, for 2 days. This protocol yielded a total of 96 sampling events, corresponding to four trap events per field in each month. For the seasonal trend analyses, abundances were standardised by sampling effort and expressed as the number of individuals per trap per day. Air temperature during the sampling period was obtained from official meteorological stations. The general trap deployment scheme and exposure procedures are consistent with those used in related Azorean dung-beetle sampling studies (Duenas-Rojas et al. 2025).

Although the traps were baited with fresh cow dung to target coprophagous insects, baited pitfall traps also sample a broader fraction of ground-active arthropods, including taxa not directly associated with dung, whose capture reflects epigean activity, dispersal and occasional short-range attraction to bait odours. Therefore, order-level patterns, based on total trap catches, should be interpreted as activity-density in dung-baited traps rather than as strict dung association.

### Quality control

After collection, samples were stored in ethanol (96%) before sorting. Specimens, adults and juveniles, were identified following a system of morphospecies by a trained parataxonomist (Abrão Leite) and final identification was done by the senior author (Paulo A.V. Borges). Nomenclature of the species follows Borges et al. (2022).

## Geographic coverage

### Description

Terceira Island, Azores, Portugal.

### Coordinates

38.638 and 38.814 Latitude; -27.394 and -27.0150 Longitude.

## Taxonomic coverage

### Description

Kingdom: Animalia;

Phylum: Arthropoda;

Class: Arachnida, Chilopoda, Diplopoda, Insecta;

Order: Araneae, Coleoptera, Dermaptera, Hemiptera, Hymenoptera, Julida, Lepidoptera, Lithobiomorpha, Opiliones, Orthoptera, Polydesmida, Psocodea, Pseudoscorpiones, Scolopendromorpha, Scutigeromorpha, Thysanoptera.

## Temporal coverage

### Notes

2022-10-14 / 2023-09-18

## Collection data

### Collection name

Dalberto Teixeira Pombo

### Collection identifier

DTP

### Specimen preservation method

Ethanol 96%

## Usage licence

### Usage licence

Creative Commons Public Domain Waiver (CC-Zero)

## Data resources

### Data package title

Dung-associated arthropods from cattle dung-baited pitfall traps across seasons, Terceira Island (Azores, Portugal)

### Resource link

https://doi.org/10.15468/bzc8wn

### Alternative identifiers

https://www.gbif.org/dataset/c44bcbc1-fefc-4ac2-9366-1106c7e99aa4; https://ipt.gbif.pt/ipt/resource?r=seasonal_dung_beetles_terceira

### Number of data sets

2

### Data set 1.

#### Data set name

Event table

#### Data format

Darwin Core Archive format

#### Character set

UTF-8

#### Data format version

1.1

#### Description

The dataset was published in the Global Biodiversity Information Facility platform, GBIF (Borges et al. 2026). The following data table includes all the records for which a taxonomic identification of the species was possible. The dataset submitted to GBIF is structured as a sample event dataset that has been published as a Darwin Core Archive (DwCA), which is a standardised format for sharing biodiversity data as a set of one or more data tables. The core data file contains 96 records (eventID). This GBIF IPT (Integrated Publishing Toolkit, Version 2.5.6) archives the data and, thus, serves as the data repository. The data and resource metadata are available for download in the Portuguese GBIF Portal IPT.

**Data set 1.** 

Column label	Column description
eventID	Identifier of the events, unique for the dataset.
type	The nature or genre of the resource.
samplingProtocol	The methods or protocols used during an Event.
sampleSizeValue	A numeric value for a measurement of the size (time duration, length, area or volume) of a sample in a sampling Event.
sampleSizeUnit	The unit of measurement of the size (time duration, length, area or volume) of a sample in a sampling Event.
samplingEffort	The amount of effort expended during an Event.
eventDate	The date-time or interval during which an Event occurred.
year	Year of the event.
habitat	The habitat for an Event.
fieldNumber	Unique identifier of each trap used in the field for sampling.
locationID	Identifier of the locations, unique for the dataset.
continent	The name of the continent where the occurrence was recorded.
islandGroup	The name of the island group in which the Location occurs (Azores Archipelago).
island	The name of the island on which the Location occurs (Terceira).
country	The full, unabbreviated name of the next smaller administrative region than stateProvince (county, shire, department etc.) in which the Location occurs (Portugal).
countryCode	The standard code for the country in which the Location occurs (PT).
stateProvince	The name of the next smaller administrative region than country (state, province, canton, department, region etc.) in which the Location occurs.
municipality	The full, unabbreviated name of the next smaller administrative region than county (city, municipality etc.) in which the Location occurs.
locality	The specific description of the place.
minimumElevationInMetres	The lower limit of the range of elevation (altitude, usually above sea level), in metres.
decimalLatitude	Approximate centre point decimal latitude of the field site in GPS coordinates.
decimalLongitude	Approximate centre point decimal longitude of the field site in GPS coordinates.
geodeticDatum	Standard Global Positioning System coordinate reference for the location of the sample collection points.
coordinateUncertaintyinMetres	Uncertain value of coordinate metrics.
coordinatePrecision	Value in decimal degrees to a precision of six decimal places.
georeferenceSources	Navigation system used to record the location of sample collections.
dynamicProperties	Minimum and maximum air temperatures recorded during the event, in degrees Celsius.

### Data set 2.

#### Data set name

Occurrence Table

#### Data format

Darwin Core Archive format

#### Character set

UTF-8

#### Download URL

https://ipt.gbif.pt/ipt/resource?r=seasonal_dung_beetles_terceira

#### Data format version

V 1.1

#### Description

The dataset was published in the Global Biodiversity Information Facility platform, GBIF (Borges et al. 2026). The following data table includes all the records for which a taxonomic identification of the species was possible. The dataset submitted to GBIF is structured as an occurrence table that has been published as a Darwin Core Archive (DwCA), which is a standardised format for sharing biodiversity data as a set of one or more data tables. The core data file contains 1701 records (occurrenceID). This GBIF IPT (Integrated Publishing Toolkit, Version 2.5.6) archives the data and, thus, serves as the data repository. The data and resource metadata are available for download in the Portuguese GBIF Portal IPT.

**Data set 2.** 

Column label	Column description
eventID	Identifier of the events, unique for the dataset.
occurrenceID	Identifier of the record, coded as a global unique identifier.
licence	Reference to the licence under which the record is published.
InstitutionID	An identifier for the institution publishing the data.
institutionCode	The code of the institution publishing the data.
collectionID	An identifier for the collection or dataset from which the record was derived.
collectionCode	The acronym identifying the collection or dataset from which the record was derived.
basisOfRecord	The nature of the data record.
recordedBy	A list (concatenated and separated) of names of people, groups or organisations who performed the sampling in the field.
identifiedBy	A list of names of people, groups or organisations who assigned the Taxon to the subject.
dateIdentified	The date on which the subject was determined as representing the Taxon.
sex	The sex of the biological individual(s) represented in the occurrence.
lifeStage	The age class or life stage of the Organism(s) at the time the Occurrence was recorded.
organismQuantity	A number or enumeration value for the quantity of Organisms.
organismQuantityType	The type of quantification system used for the quantity of organisms.
identificationRemarks	Dalberto Teixeira Pombo (DTP) collection's morphospecies number attributed to specimens identified.
scientificName	The full scientific name, with authorship and date information if known.
taxonRank	Lowest taxonomic rank of the record.
kingdom	Kingdom name.
phylum	Phylum name.
class	Class name.
order	Order name.
family	Family name.
genus	Genus name.
specificEpithet	Specific epithet name.
infraspecificEpithet	Name of the lowest or terminal infraspecific epithet of the scientific name.
scientificNameAuthorship	The authorship information for the scientificName formatted according to the conventions of the applicable nomenclaturalCode.
establishmentMeans	The process of establishment of the species in the location, using a controlled vocabulary: 'native', 'introduced', 'endemic', 'uncertain".

## Additional information

In total, the survey yielded 13,882 individuals distributed across four classes, 16 orders and 61 families. These specimens were assigned to 175 morphospecies, 32 of which could only be identified at order, family or genus level (1017 specimens). Consequently, 143 morphospecies were identified with full scientific name, representing 12,865 specimens (see Suppl. material 1).

**Figure 3.**
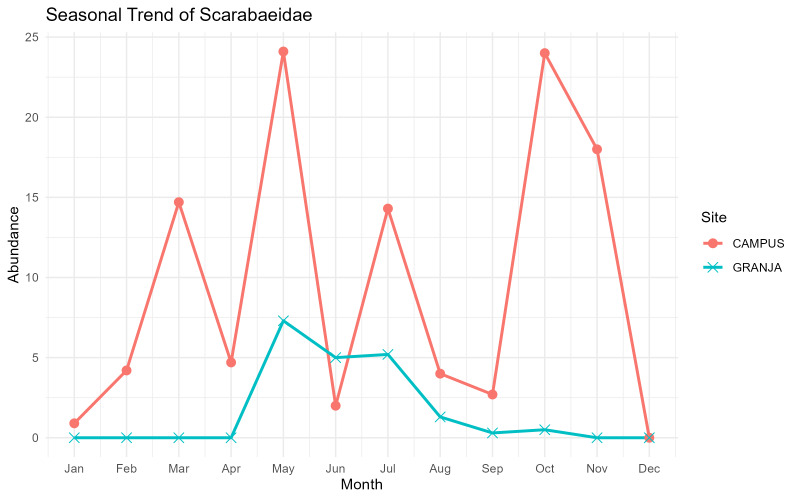
Monthly abundance trends of the Scarabaeidae in the two sampling sites, Campus and Granja, on Terceira Island, Azores.

Seasonal activity patterns differed markedly amongst the focal dung-associated groups. For Scarabaeidae, dung beetle abundance at the mid-elevation Granja site showed a clear, short activity peak between May and July, whereas, at the low-elevation University Campus, site activity was more prolonged and irregular, with several pronounced peaks from March to November and little or no activity recorded in winter (Fig. 3).

**Figure 4.**
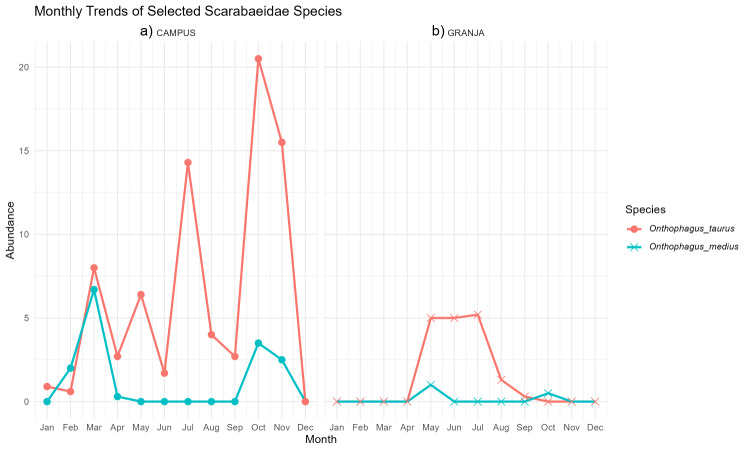
Monthly abundance of the two *Onthophagus* species (*O. taurus* and *O. medius*) at the two sampling sites. Panel (a) shows Campus and panel (b) shows Granja.

In both sites, the two exotic *Onthophagus* species found showed contrasting seasonal patterns. At the low-elevation Campus (panel A in Fig. 4), *O. taurus* was almost continuously active from winter to late autumn, with marked abundance peaks in early spring (March), mid-summer (July) and again in autumn (October–November). In contrast, *O. medius* (this species was previously known from the Azores as *O. vacca*, Duenas-Rojas et al. (2025)) was always less abundant and showed a more pulsed phenology, with a sharp peak in late winter–early spring (February–March) and a smaller resurgence in autumn (October–November). At the cooler mid-elevation Granja site (panel B in Fig. 4), *O. taurus* had a much shorter flight season, being virtually absent in winter and concentrated between May and July with a rapid decline towards early autumn, while *O. medius* occurred only sporadically, with a few individuals in late spring and autumn. Overall, *O. taurus* exhibits a longer and more extended activity period, especially at the warmer low-elevation site, whereas *O. medius* remains scarce and more seasonally restricted, patterns that agree with phenological observations for these species in other Western Palaearctic pasture systems (Roessner et al. 2010).

**Figure 5.**
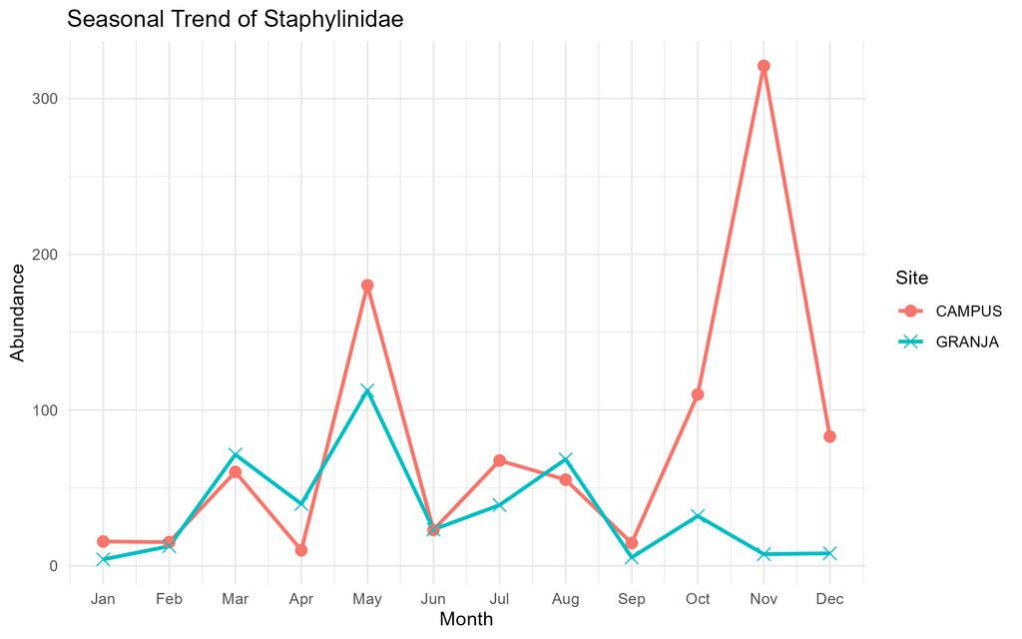
Monthly abundance trends of the Staphylinidae family in the two sampling sites, Campus and Granja, on Terceira Island, Azores.

Staphylinid (rove beetle) activity was high and extended through most of the year at both sites, but with marked differences in the timing and magnitude of peaks. At the low-elevation Campus pasture, abundances increased from winter to an early-spring maximum in March, then rose sharply to a very pronounced peak in May, followed by lower summer numbers and a second major peak in November (Fig. 5). At the mid-elevation Granja site, Staphylinidae also showed strong spring activity (March–May), but their summer activity was more sustained, with a secondary peak in August and moderate abundances persisting into early winter. In both pastures, catches dropped to a minimum in September.

**Figure 6.**
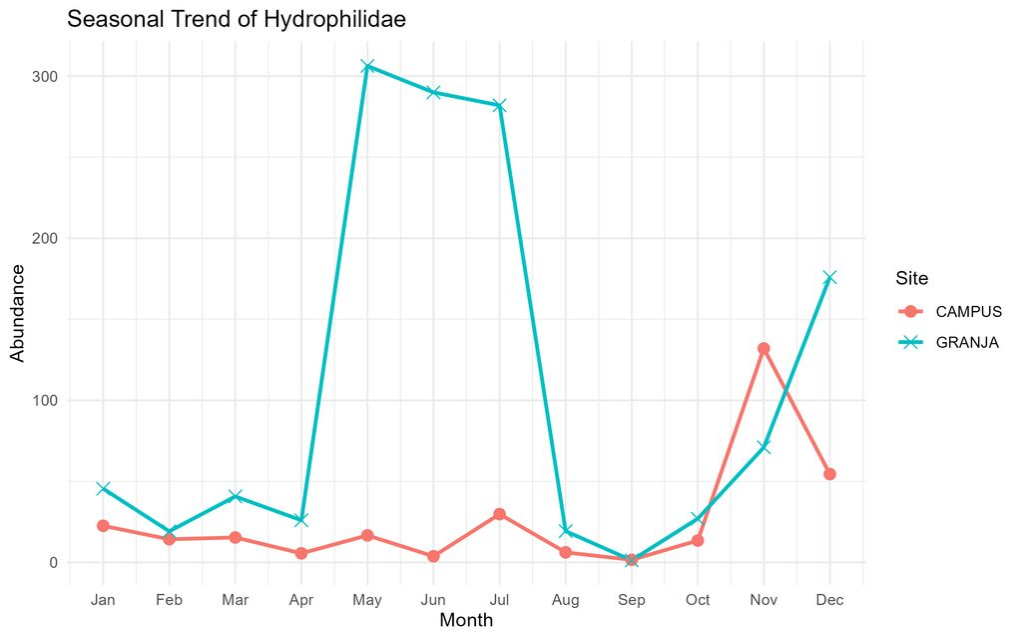
Monthly abundance trends of the Hydrophilidae family in the two sampling sites, Campus and Granja, on Terceira Island, Azores.

Hydrophilid beetles showed strikingly different seasonal dynamics between the two pastures on Terceira (Fig. 6). At the low-elevation Campus site, abundances remained relatively low to moderate throughout most of the year, with a modest spring increase (March–May), a short mid-summer rise in July and a pronounced peak in late autumn (November), followed by a decline in December. In contrast, at the mid-elevation Granja site, the family displayed a very marked and concentrated activity period: numbers increased sharply from April, reached extremely high values between May and July and then collapsed to almost zero by September, with only moderate recovery in late autumn and early winter. This strong late spring–summer peak at Granja and the weaker, more prolonged pattern at Campus are consistent with the known dependence of many coprophilous Hydrophilidae on moist dung and cooler, humid microclimates, which tend to be more favourable at mid-elevation pastures during early summer than in the drier lowlands (Mroczkowski et al. 2020).

**Figure 7.**
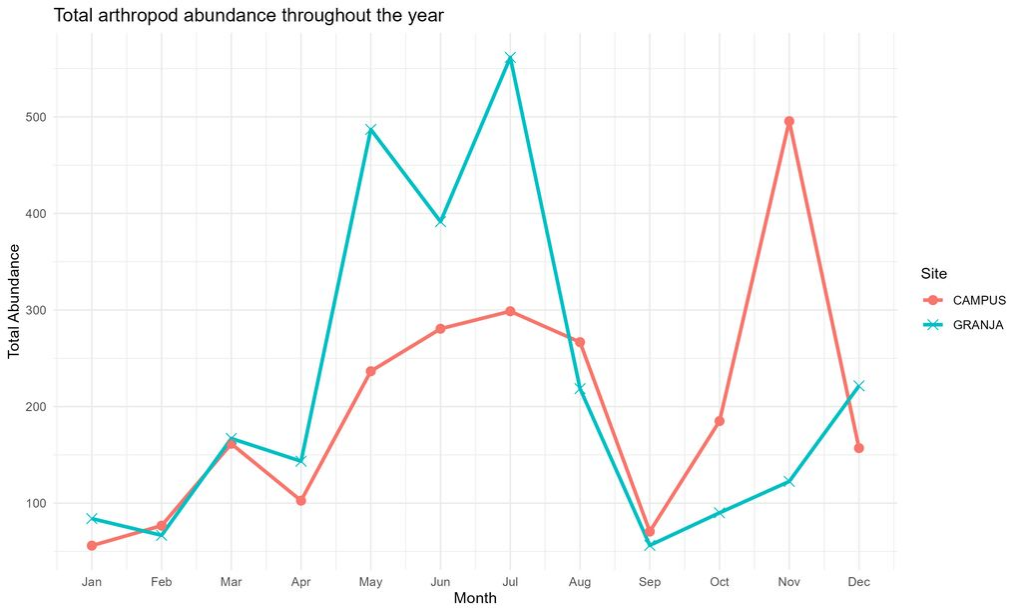
Seasonal trends in total arthropod abundance at the Campus and Granja sampling sites, Terceira Island, Azores.

Total arthropod abundance showed strong month-to-month variation and clear differences between sites (Fig. 7). At Granja, abundance rose sharply from April into late spring/early summer, with pronounced peaks in May–July, followed by a steep decline through August–September and a gradual increase towards December. At Campus, abundance increased from late winter into spring–summer (May–July), dropped markedly in September and then displayed a distinct late-autumn peak in November before declining again in December. Overall, both sites shared a common minimum around September, but Granja exhibited a stronger spring–summer pulse, whereas Campus showed a comparatively larger late-autumn resurgence, consistent with site-specific differences in seasonal resource availability and local conditions affecting epigean and dung-associated arthropod activity. Seasonal shifts in temperature/moisture and resource continuity are well known to structure arthropod phenology and abundance in temperate systems, including Azorean arthropod communities (Borges et al. 2017).

**Figure 8.**
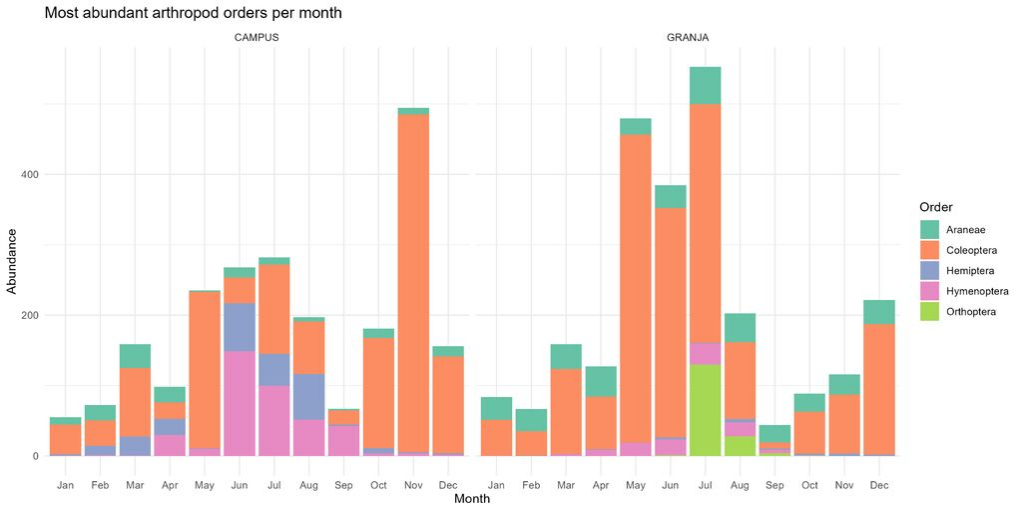
Most abundant arthropod orders captured in dung-baited pitfall traps per month at the Campus and Granja sampling sites, Terceira Island, Azores.

The Fig. 8 summarises the five most abundant arthropod orders captured each month in dung-baited pitfall traps at the two pasture sites. These data include not only taxa directly associated with dung, but also other epigean arthropods intercepted during ground-surface movement or dispersal; thus, the figure represents the broader trap-captured assemblage rather than exclusively dung-dependent fauna. Across the year, Coleoptera dominate total abundance at both sites and account for the main seasonal peaks. At Granja, beetle abundance increases sharply in late spring and summer, reaching very high values in May–July, followed by a marked decline in September–October and a modest recovery towards winter. At Campus, the monthly pattern is more irregular, with moderate values through spring and summer and a pronounced late-autumn peak in November, again largely driven by Coleoptera, followed by a decline in December. The remaining orders contribute less to total abundance, although Araneae show occasional increases and Orthoptera make a clear contribution at Granja in mid-summer.

As Campus and Granja differ simultaneously in elevation and pasture management, the contrast shown in this figure should be interpreted as a site-level descriptive comparison rather than as evidence of a pure altitudinal effect. In particular, the more regular spring–summer peak at Granja is likely related to continuous grazing and greater temporal continuity of dung resources, whereas the more irregular pattern at Campus probably reflects the seasonal interruption of grazing, superimposed on local climatic differences associated with elevation. This interpretation is ecologically plausible, since both insect communities in general and dung beetle assemblages in particular are known to respond to elevational climatic gradients, while dung-resource continuity and habitat context strongly influence abundance and diversity patterns.

### Concluding remarks and future research agenda

The annual series highlights how seasonality and local management interact to structure dung-associated assemblages on an oceanic island with a comparatively small, exotic-influenced species pool. In particular, focal beetle families show contrasting phenologies between the continuously grazed mid-elevation pasture (Granja) and the low-elevation pasture with a summer grazing interruption (Campus), consistent with the expectation that resource continuity (dung availability) can modulate seasonal activity peaks superimposed on climatic drivers.

By providing openly reusable, event-centred records aligned with biodiversity standards, the dataset supports robust comparisons amongst sites, guilds and months and provides a baseline for tracking how climate variability and pasture management may reshape the timing and dominance of key decomposer and predator groups that underpin dung removal, nutrient cycling and pest suppression in grazed landscapes.

This first annual baseline dung associated arthropods can be extended in two especially valuable directions: (i) multi-year continuation to quantify interannual variability and detect directional shifts in phenology consistent with warming or altered rainfall regimes and (ii) integration with functional traits and ecosystem functions (e.g. dung removal assays, soil nutrients, fly suppression) to directly link seasonal community turnover to ecosystem service delivery. These steps align well with trait-based dung beetle ecology frameworks and broader biodiversity–ecosystem functioning (BEF) tests across continental vs. insular species pools (deCastro‐Arrazola et al. 2022).

## Supplementary Material

4E1408E3-34F5-5BC1-97BB-2EB28406A8C0 10.3897/BDJ.14.e186171.suppl1 Supplementary material 1 List of identified species and subspecies Data type Excel (XLS) Brief description The detailed list of species with indcation of the establishment means (endemic, native, introduced, uncertain). File: oo_1561689.xlsx https://binary.pensoft.net/file/1561689 Paulo A. V. Borges
